# Detection of antibiotic-resistant bacteria and their resistance genes from houseflies

**DOI:** 10.14202/vetworld.2020.266-274

**Published:** 2020-02-12

**Authors:** Sharmin Akter, Abdullah Al Momen Sabuj, Zobayda Farzana Haque, Md. Abdul Kafi, Md. Tanvir Rahman, Sukumar Saha

**Affiliations:** Department of Microbiology and Hygiene, Faculty of Veterinary Science, Bangladesh Agricultural University, Mymensingh-2202, Bangladesh

**Keywords:** antibiotic resistance genes, antibiotic-resistant bacteria, houseflies

## Abstract

**Background and Aim:**

Houseflies (*Musca domestica*) are synanthropic insects which serve as biological or mechanical vectors for spreading multidrug-resistant bacteria responsible for many infectious diseases. This study aimed to detect antibiotic-resistant bacteria from houseflies, and to examine their resistance genes.

**Materials and Methods:**

A total of 140 houseflies were captured using sterile nylon net from seven places of Mymensingh city, Bangladesh. Immediately after collection, flies were transferred to a sterile zipper bag and brought to microbiology laboratory within 1 h. Three bacterial species were isolated from houseflies, based on cultural and molecular tests. After that, the isolates were subjected to antimicrobial susceptibility testing against commonly used antibiotics, by the disk diffusion method. Finally, the detection of antibiotic resistance genes *tetA*, *tetB*, *mcr-3*, *mecA*, and *mecC* was performed by a polymerase chain reaction.

**Results:**

The most common isolates were *Staphylococcus aureus* (78.6%), *Salmonella* spp., (66.4%), and *Escherichia coli* (51.4%). These species of bacteria were recovered from 78.3% of isolates from the Mymensingh Medical College Hospital areas. Most of the isolates of the three bacterial species were resistant to erythromycin, tetracycline, penicillin and amoxicillin and were sensitive to ciprofloxacin, ceftriaxone, chloramphenicol, gentamicin, and azithromycin. Five antibiotic resistance genes of three bacteria were detected: *tetA*, *tetB*, *mcr-3*, and *mecA* were found in 37%, 20%, 20%, and 14% isolates, respectively, and no isolates were positive for *mecC* gene.

**Conclusion:**

*S. aureus*, *Salmonella* spp., and *E. coli* with genetically-mediated multiple antibiotic resistance are carried in houseflies in the Mymensingh region. Flies may, therefore, represent an important means of transmission of these antibiotic-resistant bacteria, with consequent risks to human and animal health.

## Introduction

Vector-borne diseases have become a global public health concern that is directly related to human health and with the propensity to significantly affect the economy of a country. Several vectors, such as houseflies, mosquitoes, ticks, sand flies, mites, lice, snails carry bacteria, viruses, and parasites, are capable of causing vector-borne illness in humans [1]. According to the WHO [1], major vector-borne diseases are responsible for around 17% of all infectious diseases globally per year, with a particularly high prevalence in tropical and subtropical areas.

The housefly (*Musca domestica*) of Muscidae family and *Diptera* order, is known as one of the most abundant and important vectors for devastating diseases of humans and animals [2]. It is frequently and persistently found in human and animal habitats, i.e., animal manure, bedding materials, household kitchens, restaurants, hospitals, dust bins, canteen, and decaying vegetable matter where they reproduce and develop their life cycle [3,4]. The fly usually carries pathogenic micro-organisms by attaching them in their mouth, wings, foot, and body surface, and also through regurgitation of gut contents [5]. Several species of bacteria have been recovered from houseflies, including *Staphylococcus aureus*, *Salmonella* spp., *Escherichia coli*, *Shigella* spp., *Campylobacter* spp., *Pseudomonas* spp., *Vibrio* spp., *Bacillus* spp., and *Enterococcus faecalis* [6-11]. Apart from annoying the animal or human, the presence of these bacteria in flies has been implicated in spreading associated diseases, such as enteric fever, anthrax, shigellosis, cholera, tuberculosis, diarrhea, from human to human, human to animal, and animal to human [12].

Antimicrobial resistance has become a serious issue over recent years throughout the world, and day by day, new resistance mechanisms are emerging and being discovered within the micro-organisms [13]. Antibiotics of the β-lactamase group are most commonly used in staphylococcal infection in humans and animals but, due to a high level of resistance to these and other antibiotics, it becomes increasingly difficult to treat such infections [14]. Methicillin-resistant *S. aureus* (MRSA), currently recognized as a “superbug,” is resistant to almost every available antibiotic [15]. MRSA is increasingly recognized as a problematic pathogen in environmental settings, carry antibiotic resistance genes such as *mecA* and *mecC* [16]; and MRSA with resistance genes such as *mecA* and *nuc* is frequently isolated from human, animal, environmental, and food samples in different parts of Bangladesh [17-20]. Similarly, colistin is frequently used in poultry in Bangladesh, although it is the reserved group of antibiotics [21]. Colistin resistance genes (*mcr-1*, *mcr-2*, *mcr-3*, *mcr-4*, and *mcr-5*), which appear to be newly developed antibiotic resistance genes, are frequently found in bacteria in the environment [22] and, recently, have been recovered from various environmental samples such as poultry, houseflies, pond water, and sludge samples in Bangladesh [23,24]. Furthermore, in Bangladesh, bacteria with the tetracycline resistance genes *tetA*, *tetB*, *tetC*, and *tetD* are frequently isolated from the gut of humans, as well as from dairy farms and environmental settings [23,25].

Marshall *et al*. [16] first showed that houseflies can disseminate antibiotic resistance genes among animals. Houseflies can easily pick up antibiotic-resistant bacteria and transmit them to and between humans and animals [2]. Several recent studies have been undertaken to examine the role of houseflies in relation to the dissemination of antibiotic resistance [26-28].

Houseflies are very common insects and frequently found in Bangladesh as the weather permits favorable conditions for their survival [29]. Several studies have been conducted in Bangladesh related to isolation and antibiogram pattern of bacteria from flies [29-31].

However, there is very limited information on the presence of antibiotic resistance genes of bacteria carried by houseflies in Bangladesh. This study aimed to detect antibiotic-resistant bacteria and their resistance genes from housefly present in Mymensingh city, Bangladesh.

## Materials and Methods

### Ethical approval and informed consent

No ethical approval was required since the research does not contain any studies with human and animal subjects. However, verbal permission was taken from the concerned authorities during the collection of samples.

### Sample collection and processing

A total of 140 houseflies were collected independently using sterile nylon nets from seven different locations, including households, restaurants, the university canteen, veterinary teaching hospital, poultry farms, dairy farms, and Mymensingh Medical College Hospital (MMCH) during the period from July to December 2018, 20 for each location. All the study locations were situated in and around Bangladesh Agricultural University campus and Mymensingh Sadar areas, Mymensingh, Bangladesh (24.45°N, 90.24°E). These places were selected on the basis of fly abundances, condition favorable for their survival, and persistent human movements. Immediately after collection, flies were transferred to a sterile zipper bag from the capture nylon net and brought to the microbiology laboratory within 1 h. Flies were then stored in these zipper bags at −20°C freezer until further processing. Then, flies were identified morphologically using stereo-microscope to ensure that they were *M. domestica* [32]. Each fly was placed, using sterile forceps, in a 15 ml Falcon tube containing phosphate-buffered saline (PBS) solution and vigorously agitated. Thereafter, 1 ml PBS solution was transferred in a test tube containing nutrient broth, after which it was incubated for 6-8 h at 37°C for enrichment.

### Isolation and identification of bacteria

For isolation, a sterile loop was used to inoculate the culture broth onto mannitol salt (MS) agar, xylose lysine deoxycholate (XLD) agar, and eosin methylene blue (EMB) agar (HiMedia, Mumbai, India) culture media. After incubation at 37°C for 24-48 h, colonies that were golden yellow, black center, or a metallic sheen in MS, XLD, and EMB agar, respectively, were identified as presumptive *S. aureus*, *Salmonella* spp., and *E. coli* [33]. Further morphological identification was done through Gram’s staining and biochemical confirmation (sugar fermentation, methyl red, Voges–Proskauer, catalase, and coagulase test) [34]. Presumptive isolates were finally confirmed by polymerase chain reaction (PCR) using previously published genus-specific oligonucleotide primers [35-37].

### Antimicrobial susceptibility test

All the isolates of the three bacteria under investigation were tested for antimicrobial susceptibility test by disk diffusion test [38], using ciprofloxacin (5 µg), ceftriaxone (30 µg), chloramphenicol (30 µg), gentamycin (10 µg), azithromycin (30 µg), amoxicillin (30 µg), nalidixic acid (30 µg), streptomycin (10 µg), erythromycin (5 µg), tetracycline (30 µg), penicillin (10 µg), and colistin (10 µg) (HiMedia, Mumbai, India). In all bacterial isolates, 0.5 McFarland suspensions were used as a standard to equalize the turbidity. The zone of growth inhibition in Mueller-Hinton agar media for each isolate was measured and compared with the standards as recommended by the Clinical and Laboratory Standards Institute [39].

### Molecular detection of antibiotic resistance genes

Isolates of *S. aureus* showing phenotypic resistance to amoxicillin and penicillin, *Salmonella* spp. to tetracycline, and *E. coli* to colistin were further tested for the detection of antibiotic resistance genes, namely, *mecA*, *mecC*, and *tetA*, *tetB*, and *mcr-3*, respectively. Antibiotic resistance genes were determined by PCR using established primers [40-43], as illustrated in Table-1.

**Table-1:** List of primers used in this study with sequences.

Target genes	Primer sequences (5’-3’)	Amplicon size (bp)	References
*Staphylococcus aureus*	F-GGAGGAAGGTGGGGATGACG R-ATGGTGTGACGGGC GGTGTG	241	[35]
*Salmonella* spp. 16S rRNA	F-ACTGGCGTTATCCCTTTCTCTGGTG R-ATGTTGTCCTGCCCCTGGTAAGAGA	496	[36]
*Escherichia coli* 16S rRNA	F-AATTGAAGAGTTTGATCATG R-CTCTACGCATTTCACCGCTAC	704	[37]
*mecA*	F-AAAATCGATGGTAAAGGTTGG R-AGTTCTGGCACTACCGGATTTTGC	533	[40]
*mecC*	F-GAAAAAAAGGCTTAGAACGCCTC R-GAAGATCTTTTCCGTTTTCAGC	138	[41]
*tetA*	F-GAAGATCTTTTCCGTTTTCAGC R-CTGTCCGACAAGTTGCATGA	577	[42]
*tetB*	F-CCTCAGCTTCTCAACGCGTG R-GCACCTTGCTGATGACTCTT	634	[42]
*mcr-3*	F- TTGGCACTGTATTTTGCATTT R- TTAACGAAATTGGCTGGAACA	542	[43]

## Results

### Prevalence of bacteria based on molecular identification

**Figure-1:**
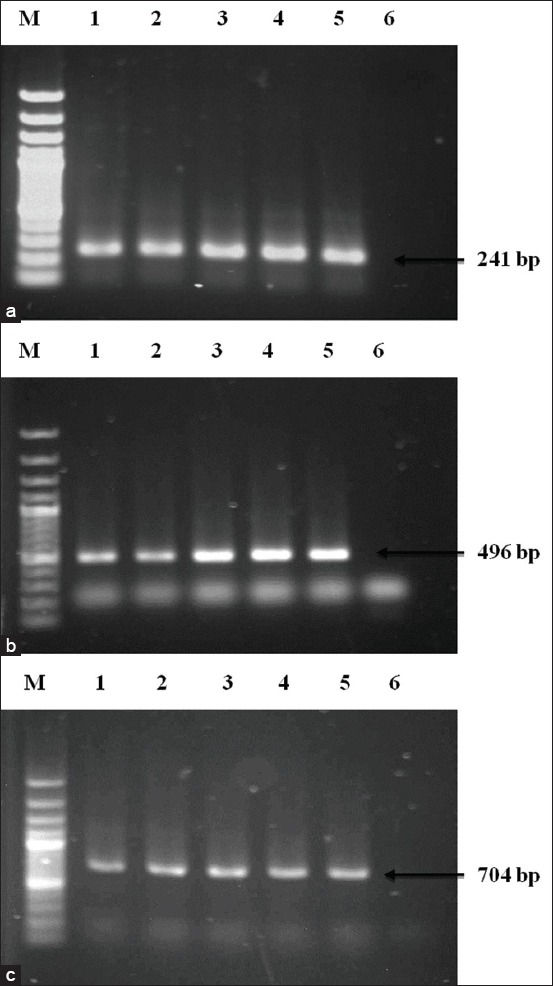
Polymerase chain reaction (PCR) amplification of 16S rRNA of *Staphylococcus aureus*, *Salmonella* spp., and *Escherichia coli* (a) PCR amplification of *S. aureus*. Lane M: 100 bp DNA Marker, 1-4: Representative *S. aureus* isolates, 5: Positive control, 6: Negative control. (b) PCR amplification of *Salmonella* spp. Lane M: 100 bp DNA Marker, 1-4: Representative *Salmonella* spp. isolates, 5: Positive control, 6: Negative control. (c) PCR amplification of *E. coli*. Lane M: 100 bp DNA Marker, 1-4: Representative *E. coli* isolates, 5: Positive control, 6: Negative control.

A total of 110 (78.6%) isolates of *S. aureus*, 93 (66.4%) isolates of *Salmonella* spp., and 72 (51.4%) isolates of *E. coli* were recovered from 140 houseflies (Table-2 and Figure-1). Among the seven sampling areas, the highest percentages of bacteria were isolated from MMCH (78.3%) and the lowest from households’ flies (48.3%).

**Table-2:** Number of bacterial isolates from houseflies.

Sampling area (n=sample size)	Number of positive bacteria			Total (%)

	*Staphylococcus aureus*	*Salmonella* spp.	*Escherichia coli*	
Households (n=20)	12	9	8	29 (48.3)
Restaurants (n=20)	17	16	13	46 (76.7)
University Canteens (n=20)	16	12	12	40 (66.7)
VTH (n=20)	18	16	8	42 (70)
Poultry farms (n=20)	12	18	8	40 (66.7)
Dairy farms (n=20)	15	8	8	31 (51.7)
MMCH (n=20)	18	14	15	47 (78.3)
Total (n=140) (%)	110 (78.6)	93 (66.4)	72 (51.4)	

VTH=Veterinary Teaching Hospital; MMCH=Mymensingh Medical College Hospital

### Antibiotic susceptibility test

Antibiotic resistance pattern of isolated *S. aureus*, *Salmonella* spp., and *E. coli* is shown in Tables-3-5. Isolates of *S. aureus* were all (100%) resistant to penicillin, followed by 94% resistant to amoxicillin and streptomycin, 93% to erythromycin, and 84% to tetracycline (Table-3). Conversely, 83% were sensitive to chloramphenicol, 80% to ceftriaxone, 78% to ciprofloxacin, and 70% to gentamycin, respectively. For *Salmonella* spp. (Table-4), isolates were resistant to erythromycin (97%), streptomycin (93%), tetracycline (90%), amoxicillin (88%), and nalidixic acid (62%), but were sensitive to chloramphenicol, ciprofloxacin, gentamycin, ceftriaxone, and azithromycin. Finally, isolates of *E. coli* were 97% resistant to erythromycin and tetracycline, 88% to streptomycin, and 85% to amoxicillin (Table-5). Fewer isolates were resistant to chloramphenicol (28%), gentamycin (28%), colistin (33%), and ciprofloxacin (38%), respectively.

**Table-3:** Antibiotic resistance pattern of *Staphylococcus aureus* isolated from houseflies.

Locations	Number of resistant isolates										

	CIP	CTR	C	GEN	AZM	AMX	NA	S	E	T	P
Households	2	3	0	2	7	12	10	12	12	11	12
Restaurants	1	2	1	2	4	16	10	12	16	17	17
University Canteens	2	1	3	4	8	16	12	16	16	12	16
VTH	6	4	2	4	4	16	10	16	16	12	18
Poultry farms	4	0	2	3	2	14	8	14	11	13	14
Dairy farms	3	5	2	6	6	13	7	15	13	9	15
MMCH	6	7	8	12	8	16	13	18	18	18	18
Total (n=110) (%)	24 (22)	22 (20)	18 (16)	33 (30)	39 (36)	103 (94)	70 (67)	103 (94)	102 (93)	92 (84)	110 (100)

CIP=Ciprofloxacin, CTR=Ceftriaxone, C=Chloramphenicol, GEN=Gentamycin, AZM=Azithromycin, NA=Nalidixic acid, S=Streptomycin, E=Erythromycin, T=Tetracycline, P=Penicillin, VTH=Veterinary Teaching Hospital, MMCH=Mymensingh Medical College Hospital

**Table-4:** Antibiotic resistance pattern of *Salmonella* spp. isolated from houseflies.

Locations	Number of resistant isolates									

	CIP	CTR	C	GEN	AZM	AMX	NA	S	E	T
Households	1	2	1	1	3	7	4	6	6	7
Restaurants	2	4	0	2	4	16	2	16	16	16
University Canteens	1	2	2	3	4	12	6	10	12	9
VTH	4	3	2	4	2	12	10	16	16	12
Poultry farms	7	8	8	3	12	18	16	18	18	18
Dairy farms	2	3	2	4	4	8	8	6	8	8
MMCH	4	10	5	8	3	9	12	14	14	14
Total (n=93) (%)	21 (23)	32 (34)	20 (22)	25 (27)	32 (34)	82 (88)	58 (62)	86 (93)	90 (97)	84 (90)

CIP=Ciprofloxacin, CTR=Ceftriaxone, C=Chloramphenicol, GEN=Gentamycin, AZM=Azithromycin, NA=Nalidixic acid, S=Streptomycin, E=Erythromycin, T=Tetracycline, VTH=Veterinary Teaching Hospital, MMCH=Mymensingh Medical College Hospital

**Table-5:** Antibiotic resistance pattern of *Escherichia coli* isolated from houseflies.

Locations	Number of resistant isolates										

	CIP	CTR	C	GEN	AZM	AMX	NA	S	E	T	CL
Households	1	2	0	1	2	8	2	6	6	8	0
Restaurants	2	1	0	3	8	10	5	13	13	13	4
University Canteens	4	3	1	3	2	10	6	9	12	12	3
VTH	2	2	3	2	4	8	4	8	8	6	3
Poultry farms	4	5	6	2	7	5	6	8	8	8	4
Dairy farms	4	3	4	3	2	8	4	4	8	8	4
MMCH	10	12	6	6	10	12	8	15	15	15	6
Total (n=72) (%)	27 (38)	28 (39)	20 (28)	20 (28)	35 (49)	61 (85)	34 (47)	63 (88)	70 (97)	70 (97)	24 (33)

CIP=Ciprofloxacin, CTR=Ceftriaxone, C=Chloramphenicol, GEN=Gentamycin, AZM=Azithromycin, NA=Nalidixic acid, S=Streptomycin, E=Erythromycin, T=Tetracycline, CL=Colistin, VTH=Veterinary Teaching Hospital, MMCH=Mymensingh Medical College Hospital

### Distribution of antibiotic resistance genes

**Figure-2:**
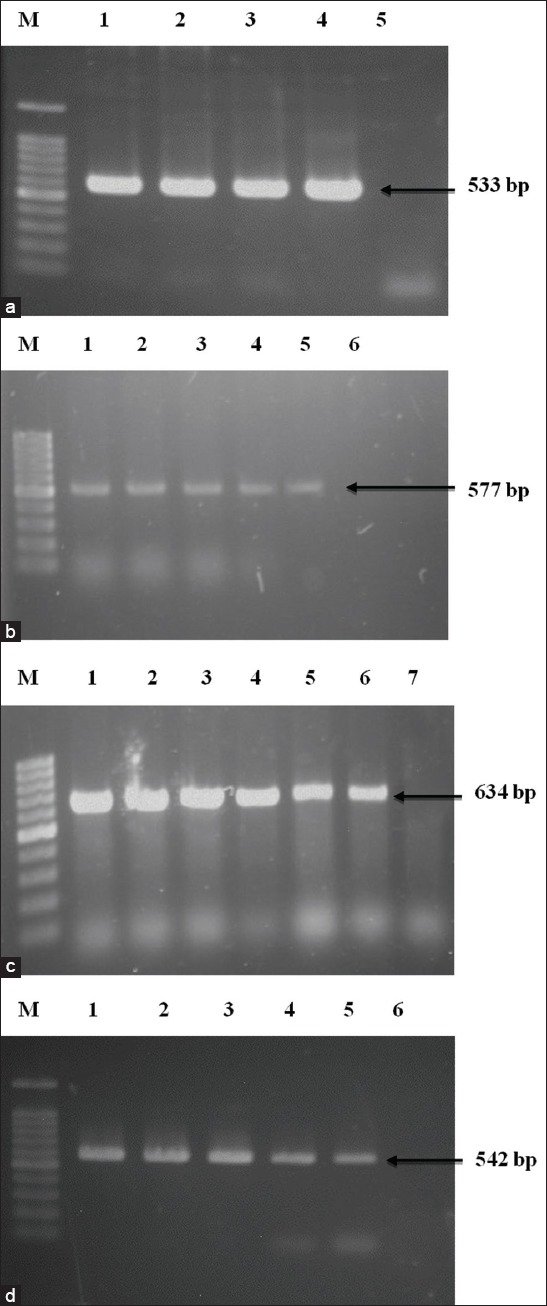
Polymerase chain reaction (PCR) amplification of antibiotic-resistant genes *Staphylococcus aureus*, *Salmonella* spp., and *Escherichia coli* (a) PCR amplification of *mecA* gene of penicillin and amoxicillin-resistant *S. aureus*. Lane M: 100 bp DNA Marker, 1-3: Representative *S. aureus* isolates, 4: Positive control, 5: Negative control. (b) PCR amplification of *tetA* gene of tetracycline-resistant *Salmonella* spp. Lane M: 100 bp DNA Marker, 1-4: Representative *Salmonella* spp. isolates, 5: Positive control, 6: Negative control. (c) PCR amplification of *tetB* gene of tetracycline-resistant *Salmonella* spp. Lane M: 100 bp DNA Marker, 1-5: Representative *Salmonella* spp. isolates, 6: Positive control, 7: Negative control. (d) PCR amplification of *mcr3* gene of colistin-resistant *E. coli*. Lane M: 100 bp DNA Marker, 1-4: Representative *E. coli* isolates, 5: Positive control, 6: Negative control.

Among the 110 isolates of *S. aureus*, 103 that were resistant to both penicillin and amoxicillin and were further investigated for the presence of *mecA* and *mecC* genes. Of 103 resistant isolates, 14% were positive for *mecA*, but all isolates were negative for *mecC*. In the case of tetracycline-resistant *Salmonella*, *tetA* was the most prevalent (44%) resistance gene, compare to *tetB* (20%). Out of 24 colistin-resistant *E. coli* isolates, 5 (20%) were positive for *mcr-3* gene (Table-6 and Figure-2).

**Table-6:** Distribution of antibiotic resistance genes of *Staphylococcus aureus*, *Salmonella* spp. and *E. coli.*

Locations	Number of penicillin and amoxicillin-resistant *Staphylococcus aureus* isolates (n=103)		Number of tetracycline-resistant *Salmonella* spp. isolates (n=84)		Number of colistin-resistant *E. coli* isolates (n=24)
		
	*mecA*	*mecC*	*tetA*	*tetB*	*mcr-3*
Households	0	0	3	1	0
Restaurants	1	0	4	2	0
University Canteens	1	0	3	0	0
VTH	2	0	6	2	1
Poultry farms	2	0	10	6	1
Dairy farms	3	0	3	2	1
MMCH	5	0	8	4	2
Total (%)	14 (14)	0 (0)	37 (44)	17 (20)	5 (20)

VTH=Veterinary Teaching Hospital, MMCH=Mymensingh Medical College Hospital, *E. coli*=*Escherichia coli*

## Discussion

The ecology and biology of the housefly (*M. domestica*) make it a potential mechanical vector for animal and human micro-organisms [4] and, indeed, it can transmit a wide range of pathogenic agents such as bacteria, virus, and fungi [44,45]. Thus, bacteria have frequently been isolated from the external surface of the fly body, as they pick up the pathogens through the mouth, legs, wings, and other body parts during the feeding process, which they carry back the pathogens to animal and human, where they complete their life cycle [46]. In the present study, three bacteria were specifically identified in houseflies, namely, *S. aurues*, *Salmonella* spp., and *E. coli*. Moreover, each fly sample carried at least one pathogen, presumably in association linked with poor hygiene and sanitation in the environment form where they are captured. Previous studies have similarly isolated *Salmonella* Typhimurium, *E. coli*, *S. aureus*, *Klebsiella*, and *Shigella* from external surfaces of houseflies circulating in a University Canteen of Dhaka, Bangladesh [29]. Seven places were included in this research from where fly samples were collected. Among them, the highest numbers of bacteria were recovered from the human hospital area (78.3%). The present findings are also comparable with the results of Nazari *et al*. [2] where higher numbers of bacterial strains were isolated from flies in hospital environments in Hamadan, Iran. In Brazil, Almeida *et al*. [47] also recovered *Staphylococcus* spp., *Salmonella* spp., and *E. coli* from both internal and external surfaces of houseflies collected from dairy farms. Ommi *et al*. [48] conducted a study in Iran on houseflies and recovered a significant number of *Salmonella* and *Campylobacter* spp. from flies from cattle farms, an animal hospital and a slaughterhouse, compared to chicken farms and human hospital. Ibrahim *et al*. [49] captured houseflies from a canteen, restaurants, and indoor food preparation premises and isolated several types of Gram-positive and Gram-negative bacteria. The frequency of *E. coli*, *Salmonella* spp., and *Staphylococcus* spp. isolations were 36.8%, 26.3%, and 42.9%, respectively, only slightly lower than in the present study. Ahmed *et al*. [50] also collected houseflies from different human and animal habitat and recovered *E. coli*, *S. aureus*, *S. albus*, *Pseudomonas aeruginosa*, *Klebsiella*, and *Salmonella*. Similar work was also done in Sokoto metropolis, Nigeria, isolated *E. coli*, *Bacillus* spp., *Pseudomonas* spp., *Staphylococcus* spp., *Enterobacter* spp., *Proteus* spp., *Salmonella* spp., and *Klebsiella* spp. [51]. Clearly, therefore, houseflies can act as a potential vector for transmitting these harmful micro-organisms through their external body surfaces and insufficient quantities [52] to cause human or animal infection.

At present, the world is facing the challenges of antibiotic-resistant bacteria, with resistance reported to most of the presently available antibiotics. It has been estimated that around 10 million people will die per year due to antimicrobial resistance by 2050 and could potentially be a significant existential threat to humans and animals [53]. Recently, several studies have been conducted on the housefly regarding antibiotic resistance and show that it plays a significant role in spreading antibiotic-resistant bacteria [2]. In the present study, micro-organisms isolated from houseflies were resistant to multiple antibiotics, and, as a matter of concern, most of the isolates displayed resistance to more than three antibiotics (namely, penicillin, amoxicillin, erythromycin and tetracycline) that are commonly prescribed in the study areas. From the seven studied areas, antibiotic-resistant bacteria were predominantly found in the human hospital areas, because this area is normally filled with sick patients who carry antibiotic-resistant bacteria, and where most of the biological products used in the hospital are simply discarded into dustbins and open places without proper treatment. Similarly, Nazari *et al*. [2] noted that organisms isolated from houseflies captured from the hospital environment showed higher resistance to antibiotics. In 2016, study of houseflies in the Dhaka district of Bangladesh found all the isolated bacteria were resistant to amoxicillin and cefixime and less resistant to chloramphenicol, gentamycin, and ciprofloxacin [29]. Another report in Chine shows that bacteria isolated from houseflies were resistant to amoxicillin, tetracycline, cefuroxime, and cephalothin [28]. Graham *et al*. [54] observed that flies captured from poultry farms carried a higher number of antibiotic-resistant bacteria and that houseflies collected from food restaurants carried a large number of antibiotic-resistant enterococci [55]. The findings of these previous studies agree with those of the present study that houseflies carry antibiotic-resistant bacteria [56-59]. An increased number of resistant bacterial strains in houseflies are almost certainly the result of frequent and haphazard use of antibiotics in human and food animals. The present results contribute to this corpus of information that houseflies carry bacteria that are resistant to multiple antibiotics, which might seriously affect human and animal health.

Antibiotic-resistant bacteria carry resistance genes that can genetically transmit to other bacteria [2]. In the present study, five antibiotic resistance genes were detected from three organisms, where *tetA* was the most common. The gene *mcr-3*, responsible for the colistin resistance, was found in 20% of *E. coli* isolates. Colistin is one of the reserve groups of antibiotics that are widely used in veterinary medicine and the agricultural sector both for treatment and prophylactic purposes. In Bangladesh, this drug has been frequently and persistently used by poultry practitioners, with the consequential appearance of resistant microorganisms. In Bangladesh, a recent study conducted by Sobur *et al*. [21] on poultry farms, houseflies, and pond water samples reported 8% colistin-resistant gene *mcr-3*. Reports are also available throughout the world for the development of colistin resistance genes. In China, Zhang *et al*. [60] also recorded the colistin resistance gene (*mcr-1*, *mcr-2*, and *mcr-3*) from houseflies and blowflies. Similarly, in Vietnam, Nguyen *et al*. [61] reported colistin resistance genes in poultry and pig farm. In the present study, MRSA gene *mecA* was found higher in number while no isolates were positive for *mecC* gene. In Bangladesh, penicillin, amoxicillin, and ampicillin are ubiquitously used in human and animal production sectors, as are methicillin and related antibiotics, with the inevitable consequence of the selection of resistant micro-organisms. Several studies in Bangladesh have looked for MRSA and their resistance genes in the dairy sector [17,62,63], but no study yet to be conducted in houseflies related to MRSA resistance genes in Bangladesh. Bacterial containing these antibiotic resistance genes can easily be picked up by houseflies from contaminated places and carried back to humans and animals, in which they can potentially cause severe infection. In addition, medical and veterinary practitioners should follow the guidelines while prescribing the antibiotics.

## Conclusion

Houseflies collected from different places carry antibiotic-resistant bacteria and their resistance genes. Therefore, regular surveillance is necessary to fully understand the significance of pathogenic bacteria carried by flies. Flies from hospital areas are contaminated with pathogenic organisms that should be controlled by a hospital authority using proper administrative procedures. In households, a fly net should be used to stop the access of flies into the kitchen. Animal and human waste and other decaying materials should be disposed of properly. Good hygienic and sanitation practices should be mandated in all restaurants and university hall canteens to minimize the fly prevalence.

## Authors’ Contributions

SA, AAMS, and ZFH carried out the research, analyzed the data, and wrote the initial draft of the manuscript. MTR contributed to manuscript writing. SS and MAK designed and supervised research work, revised, and finalized the manuscript. All authors read and approved the manuscript before submission.
